# Using Topic Modeling to Understand Patients’ and Caregivers’ Perspectives About Left Ventricular Assist Device: Thematic Analysis

**DOI:** 10.2196/50009

**Published:** 2024-08-13

**Authors:** Semyon Melnikov, Stav Klein, Moni Shahar, David Guy

**Affiliations:** 1 Nursing Department, Steyer School of Health Professions Faculty of Medical & Health Sciences Tel Aviv University Tel Aviv Israel; 2 The Tel Aviv University Center for AI and Data Science Tel Aviv University Tel Aviv Israel; 3 Steyer School of Health Professions Faculty of Medical & Health Sciences Tel Aviv University Tel Aviv Israel

**Keywords:** left ventricular assist device, LVAD, topic modeling, health care forum, heart disease, cardiovascular condition, medical devices, devices for heart, latent Dirichlet allocation, cardiovascular, device, visualization tool, tool, heart, caregiver, monitoring, management, care, users, communication, heart failure

## Abstract

**Background:**

Heart failure (HF) is a significant global clinical and public health challenge, impacting 64.3 million individuals worldwide. To address the scarcity of donor organs, left ventricular assist device (LVAD) implantation has become a crucial intervention for managing end-stage HF, serving as a bridge to heart transplantation or as a destination therapy. Web-based health forums, such as MyLVAD.com, play a vital role as trusted sources of information for individuals with HF symptoms and their caregivers.

**Objective:**

We aim to uncover the latent topics within the posts shared by users on the MyLVAD.com website.

**Methods:**

Using the latent Dirichlet allocation algorithm and a visualization tool, our objective was to uncover latent topics within the posts shared on the MyLVAD.com website. Through the application of topic modeling techniques, we analyzed 459 posts authored by recipients of LVAD and their family members from 2015 to 2023.

**Results:**

This study unveiled 5 prominent themes of concern among patients with LVAD and their family members. These themes included family support (39.5% weight value), encompassing subthemes such as family caregiving roles and emotional or practical support; clothing (23.9% weight value), with subthemes related to comfort, normalcy, and functionality; infection (18.2% weight value), covering driveline infections, prevention, and care; power (12% weight value), involving challenges associated with power dependency; and self-care maintenance, monitoring, and management (6.3% weight value), which included subthemes such as blood tests, monitoring, alarms, and device management.

**Conclusions:**

These findings contribute to a better understanding of the experiences and needs of patients implanted with LVAD, providing valuable insights for health care professionals to offer tailored support and care. By using latent Dirichlet allocation to analyze posts from the MyLVAD.com forum, this study sheds light on key topics discussed by users, facilitating improved patient care and enhanced patient-provider communication.

## Introduction

### Background

Heart failure (HF) is a serious clinical and public health problem affecting 64.3 million people worldwide [1]. With the limited availability of donor organs, management of end-stage HF has increasingly incorporated the implantation of a left ventricular assist device (LVAD) as a bridge to heart transplantation or as the destination therapy [2]. Web-based health forums have emerged as crucial and highly regarded sources of information for individuals experiencing HF or symptoms [3]. Additionally, these forums serve as valuable platforms for bridging knowledge gaps that may arise between medical consultations. They provide a space where individuals can interact with others who have similar experiences, fostering an environment that promotes enhanced understanding and mutual support [3]. Professionals concerning LVAD established MyLVAD.com, a dedicated website focused on the patient experience and care of individuals implanted with LVADs or those who are considering obtaining one [4]. The forum serves as a platform for patients with LVAD, caregivers, family members, and medical professionals to connect, share experiences, ask questions, and provide support to one another [4].

### Prior Work

In a previous study conducted by Naik et al [5], computational modeling was used to analyze discussions from web-based support groups on MyLVAD.com. The researchers used a Structural Topic Model technique to identify major topics of concern among user groups and to uncover the coping strategies used by them. The data set analyzed in this study consisted of posts from users on the MyLVAD.com forum between 2011 and 2016. Nine major topics were detected: (1) supportive and appreciative posts; (2) emotional adjustment after LVAD; (3) carrying, wearing, and handling LVAD; (4) LVADs during travel; (5) journal papers and nursing positions; (6) worried wives; (7) medical complications with LVAD; (8) food; and (9) lists of nursing notes and medical information [5]. It is important to note that the study by Naik et al [5] did not provide specific details regarding the weight of each topic or the words reservoir used to identify the topics. Additionally, the study’s findings were limited to posts collected within a specific time period.

In a subsequent study conducted by Austin et al [6], an analysis was conducted on posts, comments, and titles extracted from MyLVAD.com between April 2017 and October 2019. Using computational sentiment analysis, the study found that positive sentiment dominated the 20 most commonly used words, which indicated that, for the majority of patients and their families, LVADs and the associated experiences were viewed in a positive light [6]. Despite the positive sentiment associated with the majority of the most common words from the forum, the most common negative word in the sentiment analysis was “infection” (208 mentions), compared to other complications such as “stroke” (29 mentions), “bleeding” (30 mentions), and “thrombosis” or “clot” (32 mentions) [6].

Natural language processing (NLP) is a branch of artificial intelligence that focuses on analyzing and extracting insights from textual data [7]. Topic or text analysis is a method used to analyze large volumes of unstructured data, aiming to uncover the primary topics within each text and provide meaningful insights [8]. NLP facilitates topic analysis by automating the process through the application of various algorithms, primarily categorized into 2 main approaches: supervised and unsupervised [9]. A supervised algorithm involves the use of predetermined output attributes in addition to the input attributes, whereas an unsupervised algorithm discovers inherent groupings within unlabeled data and subsequently assigns labels to each data value [8]. The main objective of this study was to use an unsupervised algorithm and latent Dirichlet allocation (LDA), a widely used statistical modeling technique in NLP, to uncover the latent topics within the posts shared by users on the MyLVAD.com website.

## Methods

### Data Collection

**Figure 1:**
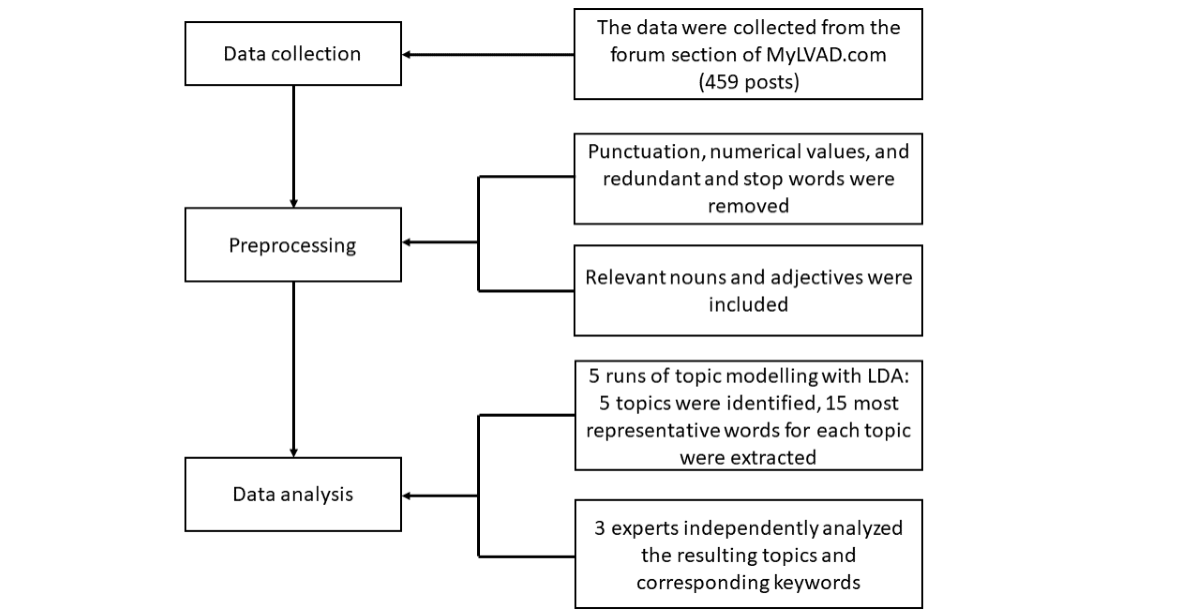
The process of data collection and analysis. LDA: latent Dirichlet allocation.

For this study, data were collected from the forum section of the website MyLVAD.com using an automated extraction method. A custom Java script was developed to systematically gather information from the forum, including thread content. The script collected all links without applying any inclusion or exclusion criteria, accessed each link, and parsed the HTML to extract the main posts, comments, and titles. The collected data were then saved in a text file and underwent preprocessing and analysis. The data set used in this study comprised 459 threads that were analyzed. Figure 1 shows the data collection and analysis process. This study did not provide compensation to participants, as it was conducted using publicly available data from a web-based forum. The research used existing data, and there was no direct interaction with individuals or collection of private information.

It is important to highlight that our extraction methods strictly followed the terms of service and policies set by the MyLVAD.com website. We were also fully cognizant of the potential risks and concerns associated with using data from web-based forums and social media platforms, such as the potential for reidentification or unintended disclosure of sensitive personal information. No personal or identifying information of forum participants was used or disclosed in this study.

### Preprocessing

The initial stage of data preprocessing aimed to enhance the quality of the data set and remove any potentially confounding elements that could impede subsequent analytical processes. Data processing included HTML formatting removal. Then, various forum-specific terms such as “author,” “comments,” “date,” “views,” and the names of days were eliminated from the data set, alongside special characters and numerical values deemed extraneous to this study’s objectives. Next, only nouns and adjectives were included using the NLP library spaCy (Explosion) [10]. Additionally, for each remaining word, its lemma (ie, its base or canonical form) was extracted using spaCy. This filtering process aimed to eliminate extraneous words and reduce the dimensionality of the data while preserving the semantic content of the text. The resulting data set comprised a more parsimonious and standardized set of features.

Before applying the LDA model to the preprocessed data, an analysis of term frequency was conducted to identify any words that may significantly skew the results of the topic modeling. In particular, frequent nouns and adjectives were examined to determine whether they would affect the topic distribution of the remaining words in the corpus. Common nouns and adjectives such as “doctor,” “patient,” and “hospital” were excluded since they were considered semantically vague and potentially detrimental to topic modeling. Additionally, words that appeared frequently throughout the data set, such as “husband,” “battery,” and “equipment,” were also flagged for exclusion due to their nondiscriminative nature concerning topic identification. The resulting data set was thus refined to include only the most salient and discriminative words, providing a more accurate representation of the underlying topics present in the corpus. The LDA analysis, without the exclusion of any words, as well as the standard list of stop words used, appears in Multimedia Appendix 1.

### Topic Modeling With LDA

LDA is a statistical modeling technique used in NLP to uncover the underlying topics present in a large corpus of textual data [7]. LDA assumes that each document in the corpus is a mixture of topics and that each topic is a distribution over words. In other words, LDA posits that there are hidden or “latent” variables, namely the topics that generate the observed words in a document. The goal of LDA is to infer the topic proportions for each document and the word probabilities for each topic by analyzing the co-occurrence patterns of words across the corpus. The LDA algorithm works by iteratively estimating the topic proportions for each document and the word probabilities for each topic until convergence. During each iteration, the algorithm randomly assigns each word in the corpus to a topic and re-estimates the topic proportions and word probabilities based on the new assignment. This process continues until the model reaches a stable state where the topic proportions and word probabilities converge [7]. The topics are then extracted from the LDA model by analyzing the estimated parameters of the model, namely the topic-word probabilities and the document-topic probabilities. The topic-word probabilities are the probabilities of each word given a particular topic. They are computed during the training phase of the LDA model and represent the relative frequency of each word in a particular topic. The document-topic probabilities, on the other hand, represent the distribution of topics in each document. These probabilities are also computed during the training phase of the LDA model and are used to determine which topics are most relevant to each document in the corpus.

To obtain robust and reliable results, the LDA model was executed on the preprocessed data for 5 runs (times). Each run was designed to identify 5 topics, and the top 15 most representative words were extracted for each topic (a complete list of the hyper-parameters used in the model, which are critical for interpreting the LDA outputs, is provided in Multimedia Appendix 2). The most representative words linked with each topic were identified based on the topic-word probabilities. Specifically, words that appeared in at least 2 of the 5 model runs were selected as the top words for each topic. This approach aimed to avoid spurious results and minimize the impact of model-specific hyper-parameters on the selected topic keywords. By selecting the most commonly occurring words across the different model runs, the resulting set of top words is considered more stable and representative of the underlying topics present in the corpus.

To validate the topics and their respective top words identified through the LDA model, 3 health care professionals (2 registered nurses with a Master of Arts and 1 author, a registered nurse with a Doctor of Philosophy) with expertise in LVAD were consulted. Further, 3 experts independently reviewed and analyzed the resulting topics and corresponding keywords. After a thorough examination, they reached an agreement regarding the validity and coherence of the identified topics, as well as their respective representative words. The consensus among the health care professionals added an important layer of verification to the LDA model results, providing further support for the accuracy and reliability of the identified topics and their associated keywords. Each topic had an assigned weight value, which was calculated by dividing the number of words in the collection assigned to the topic by the total number of words.

### Ethical Considerations

The Tel Aviv University’s ethics committee approved this study (#0006133). The ethics committee approval covers secondary analysis without additional consent.

## Results

### Overview

**Figure 2:**
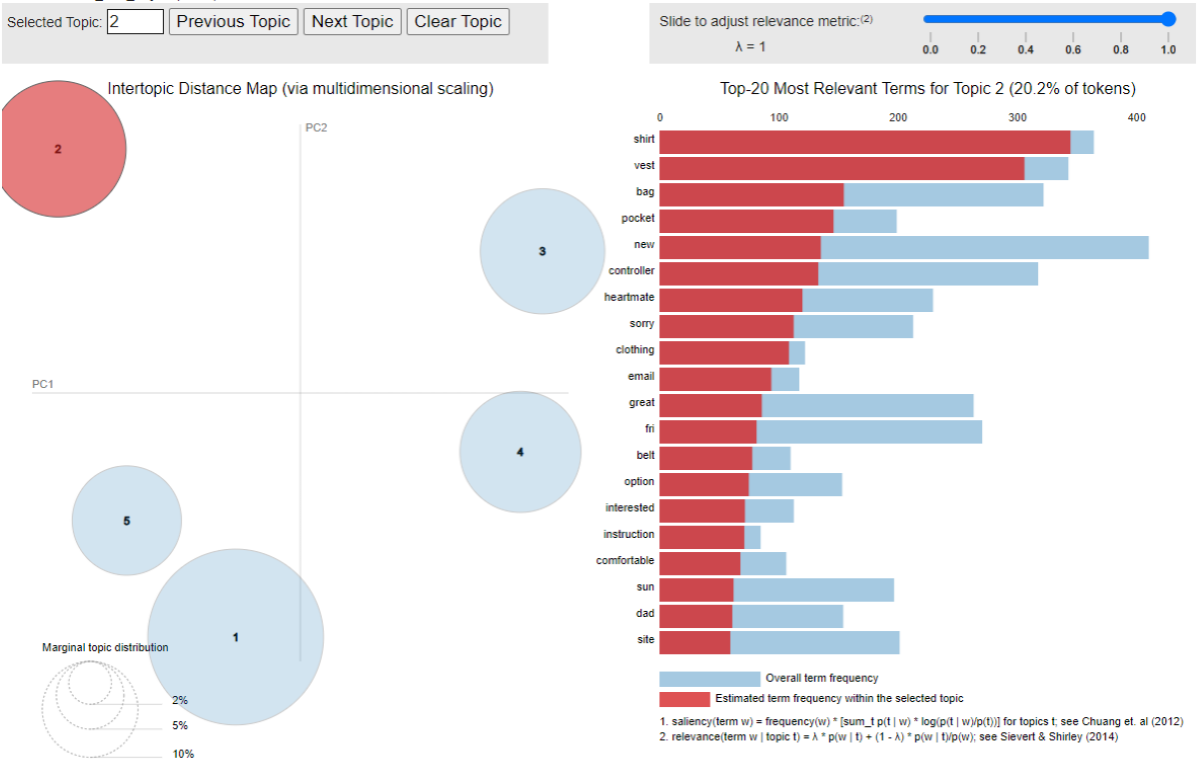
Latent Dirichlet allocation visualization of topic 2.

We identified 459 posts written by the unique users of MyLVAD.com forums from March 2015 to February 2023. A total of 945 users were identified with unique usernames. It is possible, however, that some individuals might have multiple usernames, although we lack the means to ascertain such instances. The top 5% of the most frequent users included 46 individuals. Among them, the user with the fewest posts had 14 posts, while the user with the most posts had 143 posts. It is worth mentioning that the user who ranked second in terms of post count was labeled “anonymous,” indicating those users who opted not to select a name. Further, 5 themes were identified using LDA of the posts of users of MyLVAD.com: (1) family support; (2) clothing; (3) infection; (4) power; and (5) self-care maintenance, monitoring, and management. These 5 themes with 15 keywords each are listed in Table 1. Figure 2 displays the visualizations we generated for the topics. The figure illustrates the relative importance of topics through the sizes of the circles, while the distances between the topics indicate their similarity to each other [11].

**Table 1:** The top 5 themes of concern of patients with left ventricular assist device and their family members in MyLVAD.com forum discussions.

Theme number	Themes	Keywords	Weight (%)
1	Family support	family, good, recovery, bad, able, transplant, well, infection, many, failure, new, home, post, blood, change	39.5
2	Clothing	bag, belt, clothing, comfortable, controller, pocket, vest, shirt, new, good, great, side, size, small, shoulder	23.9
3	Infection	infection, dressing, skin, change, rash, kit, tape, sterile, water, shower, bandage, area, adhesive, anchor, driveline	18.2
4	Power	bag, charger, power, extra, sure, new, good, generator, line, transplant, unit, pump, shower, water, home	12
5	Self-care maintenance, monitoring, and management	blood, controller, good, transplant, alarm, device, flow, pressure, fluid, exercise, infection, medical, rehab, dad, pump	6.3

### Family Support

The first theme was family or partner support. The weight value of this theme was 39.5%. The impact of family support was frequently mentioned in the context of post-LVAD implantation recovery. The importance of family support was mentioned both by family members as well by the patients themselves. Attributing patients’ survival to the fact that their partners or family members were by their side was frequently used. Family caregiving role: the partner (usually the wife) was portrayed as a caregiver in many posts. The partner took care of the person with LVAD, ensuring their well-being, adjusting medications, changing dressings, and maintaining precautions. The theme of caregiving and the partners’ dedication to this role was prominent. Emotional and practical support: partners not only took care of the individual’s physical needs but also provided emotional reassurance, helping patients with LVADs concentrate on becoming better. The partner’s support was seen as crucial for the successful outcome of the individual with the LVAD. Feeling like a burden: patients with LVADs expressed feelings of being useless and a burden to their partner. Family involvement: posts mentioned the involvement of the individual’s children as care providers. While the focus was primarily on the partner’s role, the presence of family members in the caregiving process was acknowledged.

### Clothing

Clothing was another major theme. The weight value of this theme was 23.9%. Some users mentioned various types of vests and shirts designed specifically for individuals with LVADs. These garments were discussed in terms of their functionality, comfort, and ability to accommodate the LVAD’s batteries and controller. Comfort and normalcy: posts emphasized the importance of finding clothing options that provided comfort and a sense of normalcy for individuals with LVADs. Wearing specific shirts, vests, or undergarments was mentioned as a way to feel more like a “normal person” despite the medical condition. Pockets and storage: users frequently mentioned the significance of pockets in LVAD clothing. Pockets are discussed in terms of their size, suitability for carrying batteries, and the convenience they offer in distributing the weight of the LVAD. Cost and bargain shopping: users provided information about where to find LVAD clothing at affordable prices, including discounted items or at specific stores. The mention of purchasing vests at marked-down prices and repurposing items from thrift stores highlighted a cost-conscious approach to acquiring suitable clothing.

### Infection

Infection was another theme we identified. The weight value of this theme was 18.2%. Driveline infections: numerous posts mentioned driveline infections as a significant concern and fear for patients with LVADs. Prevention and care: multiple posts emphasized the importance of proper care, hygiene, and proactive measures to prevent infection, such as regular dressing changes, cleanliness around the driveline, and following hospital instructions for driveline management and cable integrity. Treatment and management: the text discussed the challenges of treating infections in patients with LVADs and the use of antibiotics, blood tests, debridement, and surgeries to address infection-related issues.

### Power

The fourth theme was power. The weight value of this theme was 12%. The main subthemes were as follows. Dependence on power: the posts discussed the reliance on power for various aspects of the individual’s medical device, such as the LVAD. They emphasized the importance of maintaining a power supply and switching to battery power when necessary. Power-related challenges: multiple posts mentioned various challenges related to power, such as power failures, the need to bring personal equipment for power connection, potential risks of power switches, and the need to adapt to different power supplies when traveling. External support and coordination: numerous posts mentioned the involvement of external entities, such as LVAD coordinators, electric companies, and the fire department, in managing power-related issues. Showering precautions: there was an emphasis on taking precautions during showering to prevent water from coming into direct contact with the equipment. The posts discussed various experiences and approaches to showering while having a LVAD, including the use of protective bags, adhesive patches, and hand-held shower wands to ensure the safety of the equipment and to prevent water from reaching the device.

### Self-Care Maintenance, Monitoring, and Management

The fifth theme was self-care maintenance, monitoring, and management. The weight value of this theme was 6.3%. The following topics were identified. Blood tests and monitoring: numerous posts mentioned the importance of regular international normalized ratio blood assays, to assess the effectiveness of blood thinners. Alarms and device management: many posts mentioned the management of low flow alarms, fixes and adjustments to address alarm issues, the impact on daily life and quality of life, the importance of controller upgrades and team communication, and concerns about clots, internationally normalized ratio levels, and battery time. Physical exercises: several posts discussed finding suitable exercises and engaging in physical activities while considering the medical limitations associated with HF and LVAD. Infection prevention: numerous posts highlighted the risk, consequences, treatment, and prevention of driveline infections in patients with LVAD. Rehabilitation therapy: many posts revolved around the process of recovery and rehabilitation after a LVAD implantation, with a focus on physical rehabilitation, exercise, regaining strength, and the positive outcomes associated with rehabilitation therapy.

## Discussion

### Principal Findings

The principal findings of this innovative study, conducted through an exploration of patients’ concerns in a dedicated LVAD forum, encompassed five major themes: (1) family support plays a crucial role in the recovery and success of patients with LVAD; (2) clothing designed for users of LVADs is important for comfort and a sense of normalcy; (3) infection prevention and management, particularly driveline infections, are significant concerns for patients with LVAD, requiring proactive measures and treatment; (4) power management is essential, including maintaining power supply, dealing with power-related challenges, and involving external support for coordination; and (5) self-care maintenance, monitoring, and management involve regular blood tests, device monitoring, physical exercises, infection prevention, and rehabilitation therapy.

These findings thus advance the understanding of patients with LVAD and caregiver experiences. Our study achieved this by integrating a nuanced exploration of web-based forum discussions with innovative analytical methods. In addition to this new approach, this study also enriched the body of knowledge on LVAD-related patient concerns and needs for support in several key areas.

Insight into familial support dynamics: the theme of “family support” emerged as the most prevalent among the discussions on the MyLVAD.com forums. Previously, using Structural Topic Model analysis, Naik et al [5], identified the “supportive and appreciative posts” topic among 9 major topics in web-based support groups on MyLVAD.com. Naik et al [5] primarily highlighted major topics of concern but did not examine the specifics of familial or caregiver support. Our study thus went a step further and provided an updated contextual analysis of how family support specifically influences the recovery and daily management of patients with LVAD. This study also shed light on the critical role of emotional and practical support from family members, extending the understanding of the social dimensions of care for patients with LVAD.

A previous study by Slade et al [12] identified the cluster “partner and family support” among 9 distinct clusters encompassing the experiences of 18 recipients of LVAD from a national heart transplant center in the United Kingdom. Our study expands on this by exploring a larger and more diverse data set from the MyLVAD.com forum, offering a broader perspective on how familial support is perceived and discussed among a wider community of patients with LVAD and caregivers. The contribution of family or caregiver is expressed not only in the gratitude of the patients as has been found in this study but also by the clinical impact the family or caregiver provides. Another previous study explored the relationships between caregiver status (caregivers that retained or resigned their roles, or patients implanted with a LVAD but without an assigned caregiver) and post-LVAD implant outcomes. They found that the 30-day postimplant readmission rate was 3-fold higher for patients whose caregivers quit [13]. Lastly, another study found that following LVAD implantation, the risk of death was over 3 times more likely among individuals who lived alone compared to those who did not [14]. These findings thus support our study’s results regarding the significance and appreciation expressed by patients with LVAD toward their family members in general, and their partners in particular.

LVAD-related equipment and clothing needs: similar to this study, one of the topics identified in the MyLVAD.com users by Naik et al [5] was “carrying, wearing and handling LVAD.” Also, similar to this study, this outcome encompassed various topics such as equipment and batteries, dressings and drivelines, and the challenges of finding appropriate clothing to wear with the LVAD equipment, as found among 18 recipients of LVAD from a national heart transplant center in the United Kingdom [12]. However, the current research goes beyond only identifying the significance of clothing and equipment for patients with LVAD, as noted in such prior studies. Namely, this study explored the specific challenges and innovative solutions devised by patients to manage their LVAD equipment within their daily lives. The clothing theme not only revolved around the difficulty of finding suitable clothing to accommodate the LVAD equipment but also highlighted the patients’ desire to maintain a sense of normalcy despite their medical condition. Similarly, previous studies demonstrated that patients with LVAD adjusted their clothing not only to suit their physical needs but also to blend in with the general public [15]. It seems that despite being a lifesaving medical device worn around the clock, the user experience offered by the design of the wearable system and components of LVADs falls short of optimal. Hopefully, modern developments leading to the creation of biocompatible, lightweight, wireless LVAD devices [16] could alleviate the challenges associated with equipment and clothing. Meanwhile, clinicians can apply the current findings to offer practical advice on handling LVAD equipment in everyday life.

Another significant theme identified was “power.” This theme encompassed the concerns expressed by patients with LVAD regarding the maintenance of a reliable power supply for their LVADs, as well as the challenges they faced related to power when traveling and showering. Similarly, “LVADs during travel” was one of the major topics identified in posts from users on MyLVAD.com [5]. The current findings are also consistent with the concerns reported by patient-caregiver dyads with LVADs during cardiac rehabilitation, as highlighted in a study by Rapelli et al [17]. In that study, fear of power cuts and the need to maintain a stable power supply were among the worries expressed by patients and their caregivers. Similarly, Slade et al [12] also identified LVAD challenges as a cluster, which included patients’ concerns about equipment during shopping and the risk of getting the equipment wet. While previous research has touched upon power as a concern for patients with LVAD, this study provides a more detailed exploration of subthemes within the power category. It delves into specific aspects such as dependence on power, challenges related to power failures and traveling, the need for external support and coordination, the role of external entities such as LVAD coordinators, electric companies, and fire departments, as well as practical strategies for showering precautions. These findings collectively indicate that patients with LVADs attach great importance to a reliable power supply of their LVADs, as it represents a highly significant component of their daily lives. Based on the challenges and needs highlighted in this study, clinicians could provide ongoing self-management training for patients with LVAD to help them gain independence in maintaining a reliable power supply. Recent and potential technological advancements aimed at addressing power supply concerns for LVADs, including innovations in battery technology, wireless charging, or more energy-efficient devices [18], would help mitigate the challenges faced by patients during travel, showering, and daily activities.

“Infection” surfaced as another key theme. This finding aligns with a previous study by Austin et al [6], who found that the term “infection” was mentioned disproportionately more frequently in the MyLVAD.com forum compared to other complications. While the risk of driveline infections is a known concern, this study provides a more nuanced understanding by detailing patients’ fears, preventive measures, and the complexities involved in maintaining driveline hygiene. Currently, there is a lack of specialized training for managing cardiovascular infections in general and LVAD-associated infections in particular, despite their complexity and prevalence [19]. El-Dalati et al [19] advocated for creating programs to train health care professionals in infectious diseases, cardiology, and addiction medicine, ensuring expert, coordinated care for cardiovascular infections patients. Introducing appropriate training could potentially lower infection rates and reduce the complications from infections among patients with LVADs. At the same time, clinicians can use the insights from this study to improve patient education programs, focusing on correct driveline maintenance, identifying signs of infection, and understanding when to seek medical assistance, thereby highlighting the critical role of preventing infections.

Another significant theme identified was “self-care maintenance, monitoring, and management.” The concept of self-care is crucial in the management of chronic illnesses, as emphasized by Riegel et al [20] who defined self-care as a process that involves maintaining health through health-promoting practices and managing illness. This process comprises 3 components: self-care maintenance, self-care monitoring, and self-care management. For patients with LVADs, self-care maintenance involves behaviors aimed at maintaining physical and emotional stability [21]. These behaviors encompass operating the LVAD system, caring for percutaneous leads, and engaging in lifestyle activities such as maintaining hygiene, adhering to medications, participating in physical exercise, and following an appropriate diet. Self-care monitoring involves the ongoing monitoring of the LVAD device, symptoms and signs of HF, potential side effects and complications, as well as psychological distress. Self-care management refers to the patient’s response to symptoms and signs, which includes handling alarms, managing wound care, adjusting medications, diet, and rest, as well as seeking appropriate support from the LVAD and HF health care team [21]. Indeed, the discussions and interactions in MyLVAD.com forums show that patients and their families engage in conversations about self-care, asking questions and offering insights on various aspects of self-care management. These findings support previous results from Naik et al [5] who found “carrying, wearing, and handling LVAD,” “medical complications with LVAD,” and “food” among the major topics of posts from users on the MyLVAD.com forum. Clinicians can thus use MyLVAD.com forum discussions to develop personalized self-care plans addressing patients’ specific needs and lifestyles. Lastly, the benefits of such forums are not limited to users of LVADs. Prior research has shown that social media platforms offer social support and practical means for enabling self-care and emotional assistance to individuals living with various other medical conditions, such as those with diabetes [22].

### Limitations and Future Research

This study has several limitations. This study relied on web scraping data only from the forum section of MyLVAD.com. This introduces a potential bias, as the data collected only represents the discussions and experiences shared on this particular website. Another limitation is a sample size consisting of 459 threads. While this may be a substantial amount of data, the sample size is relatively small considering the diversity and complexity of experiences within the patients with LVAD population. A larger sample size would provide a more comprehensive understanding of the topics and themes discussed by patients with LVAD and caregivers. An additional limitation might be data generalizability. This study focuses on the forum discussions from MyLVAD.com, which may not represent the broader population of patients with LVAD and caregivers. Accordingly, the findings of this study may not be generalizable to other LVAD populations or different cultural contexts.

Future research aims to conduct a longitudinal study to track the experiences of patients with LVAD and their caregivers over an extended period of time. This would provide insights into the long-term challenges, coping strategies, and outcomes associated with LVAD implantation, in addition to a comparative analysis comparing the experiences and outcomes of patients with LVAD who participate in web-based support forums such as MyLVAD.com with those who do not. This could help determine the effectiveness and benefits of web-based support networks for patients with LVAD and identify areas for improvement.

### Conclusion

This comprehensive study, using LDA to analyze discussions from the MyLVAD.com forum, unveiled 5 pivotal themes central to the experiences of patients with LVAD: family support, clothing, infection, power, and self-care maintenance, monitoring, and management. Each theme represents a critical aspect of the patient’s journey and underscores the multifaceted nature of living with a LVAD. Recognizing these themes’ significance not only enhances the understanding but also directs their practical implications for improving patient care and support systems. The prominence of family support as the most significant theme, accounting for 39.5% of the thematic weight, underscores the indispensable role of caregivers in the postimplantation recovery phase. This finding suggests a need for health care systems to implement structured support and education programs for both patients and their caregivers, acknowledging the integral role of the family in the care continuum. The discussions around clothing and power supply challenges highlight practical aspects of daily living with a LVAD, pointing toward a need for innovations in device design and patient education that cater to these needs. The detailed exploration of infection prevention and management strategies reflects ongoing patient concerns despite existing medical guidance, indicating a gap in effective communication or the applicability of current advice. The theme of self-care maintenance, monitoring, and management reinforces the critical role of patient engagement in managing their condition. This finding suggests further investigation into the barriers and facilitators to effective self-care practices among patients with LVAD, potentially leading to the development of more intuitive and supportive self-care tools and resources.

In conclusion, this study contributes to a richer understanding of the lived experiences of patients with LVAD and the complex interplay of clinical, practical, and emotional factors that define their care journey. By illuminating these key themes, our findings provide a foundation for enhancing patient care, guiding future research, and informing the design of more responsive health care interventions for patients with LVAD and their families.

## Appendix

Multimedia Appendix 1 The latent Dirichlet allocation analysis, without the exclusion of any words, and list of stopwords.

Multimedia Appendix 2 Latent Dirichlet allocation configuration.

## Abbreviations

HF: heart failure
LDA: latent Dirichlet allocation
LVAD: left ventricular assist device
NLP: natural language processing

## Data Availability

The data sets generated and analyzed during this study are available from the corresponding author upon reasonable request.
