# Supplementary material for: Using Topic Modeling to Understand Patients’ and Caregivers’ Perspectives About Left Ventricular Assist Device: Thematic Analysis
Source: J Med Internet Res. 2024 Aug 13;26:e50009. doi: 10.2196/50009 (PMC11350299; doi:10.2196/50009)
Supplement: Multimedia Appendix 1 [file jmir_v26i1e50009_app1.docx]

The LDA analysis, without the exclusion of any words


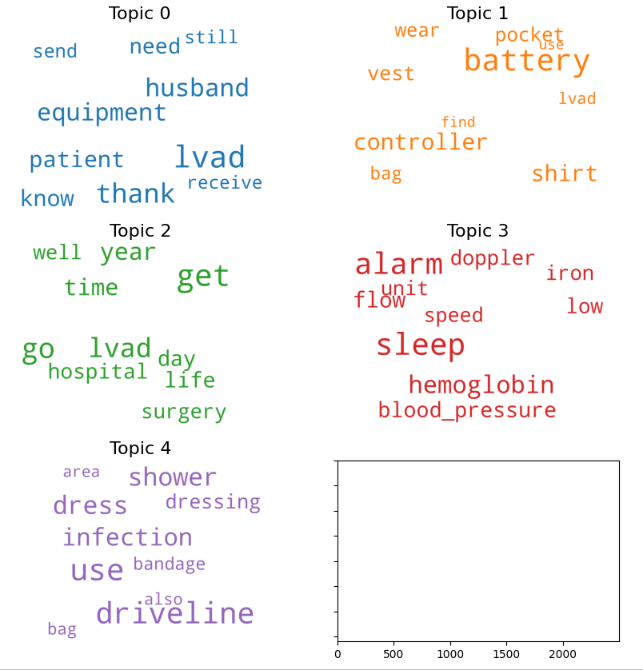

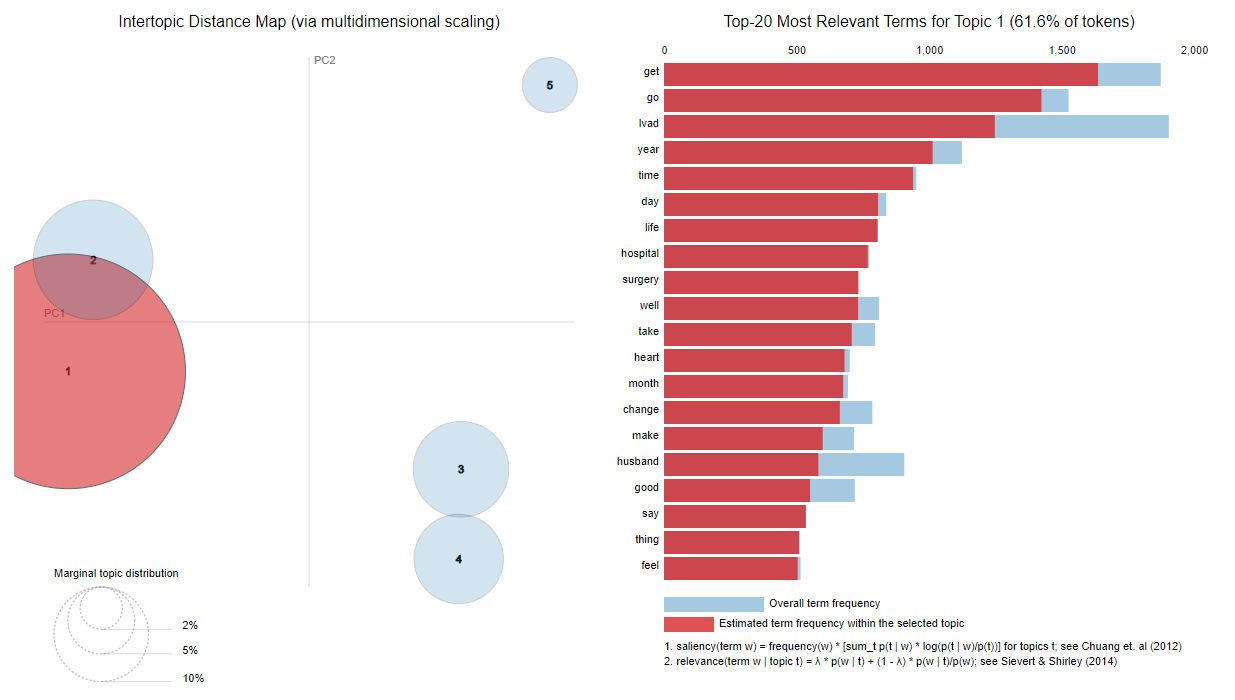

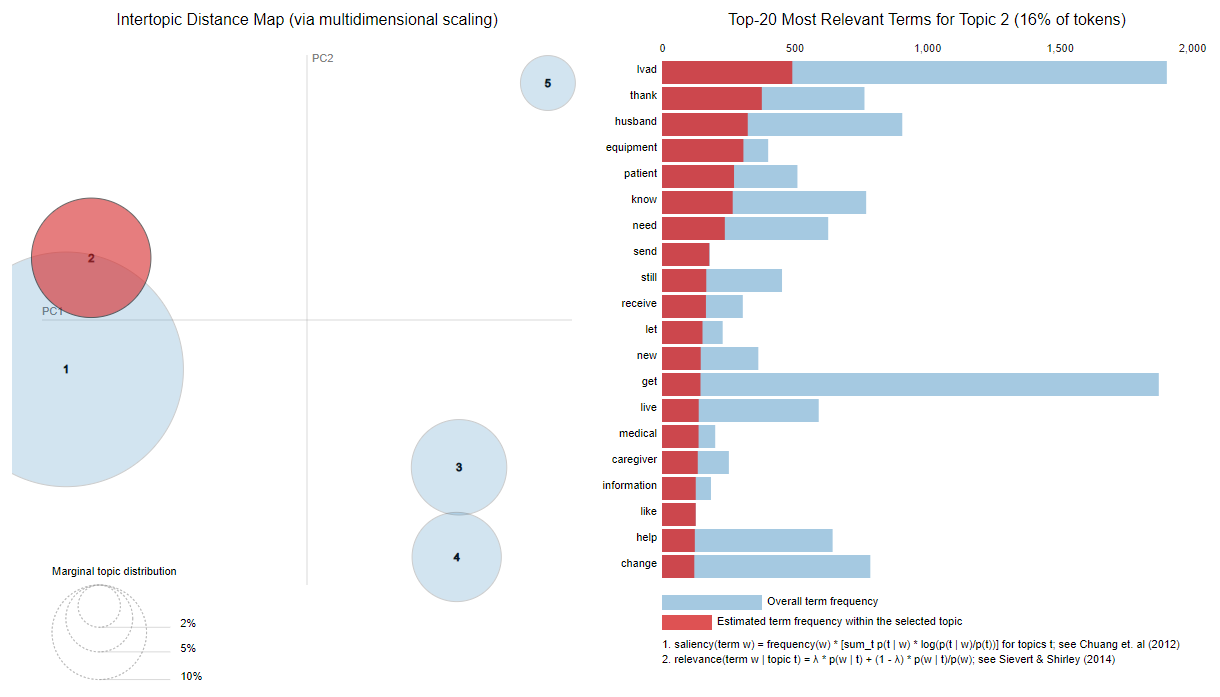

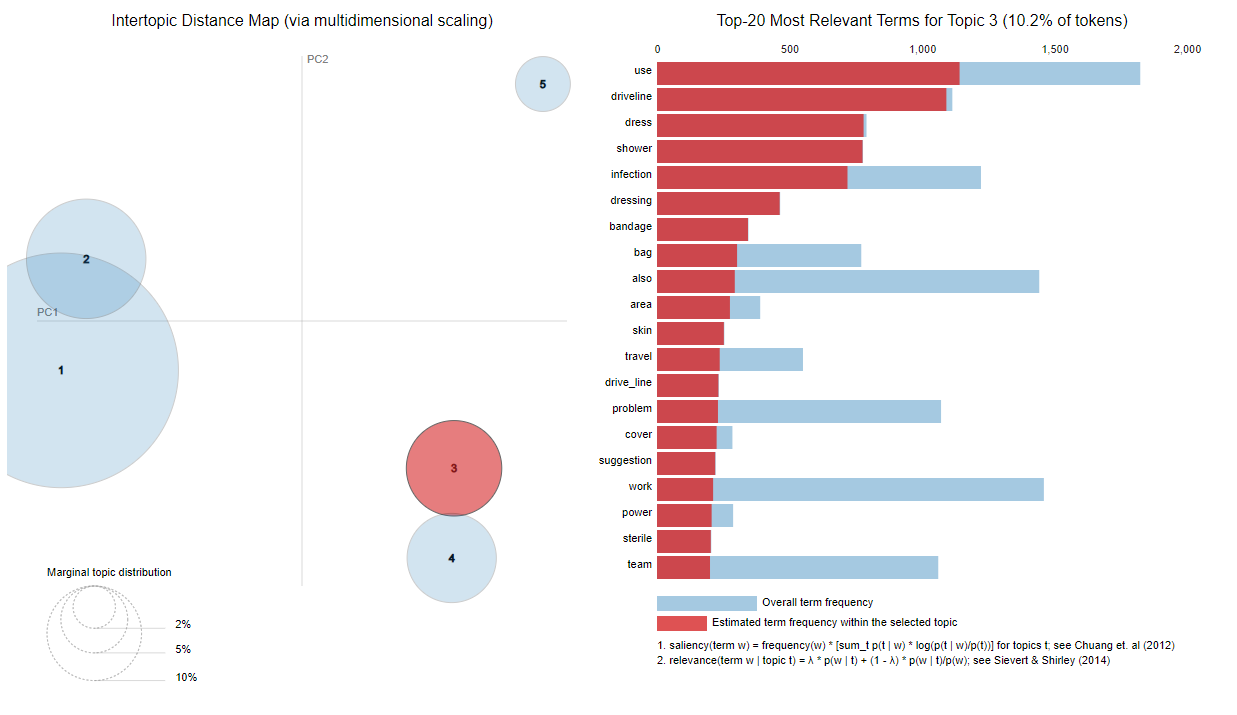

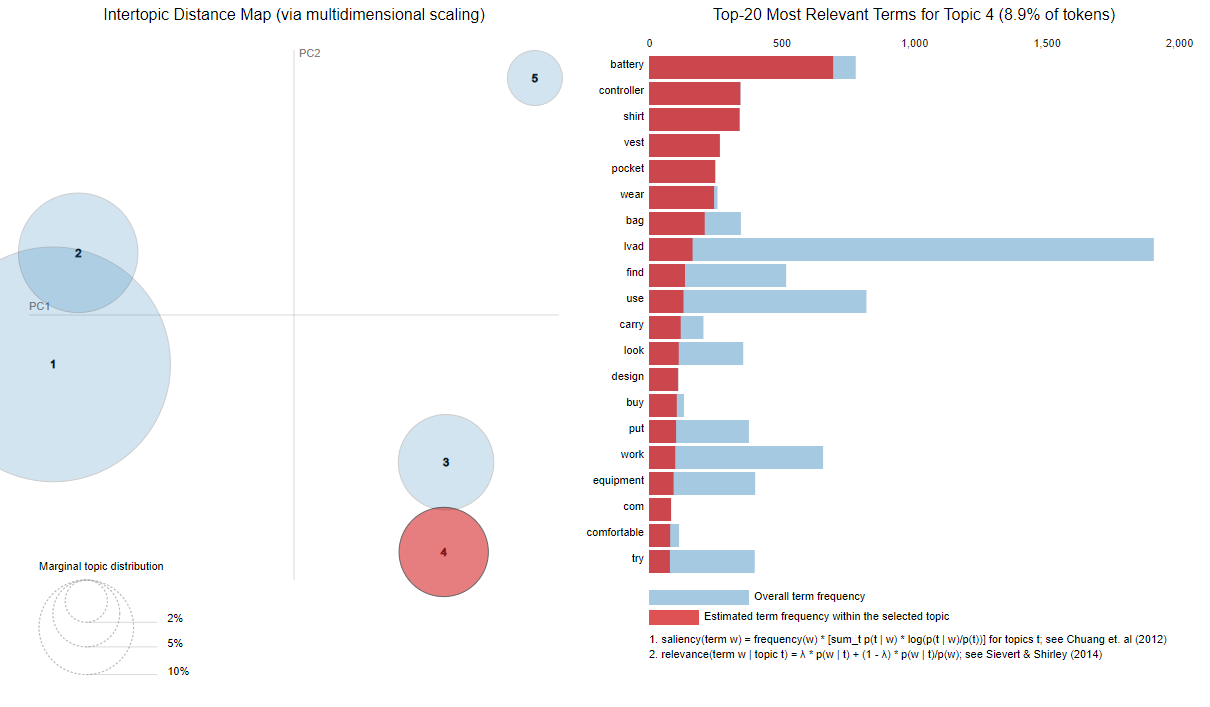

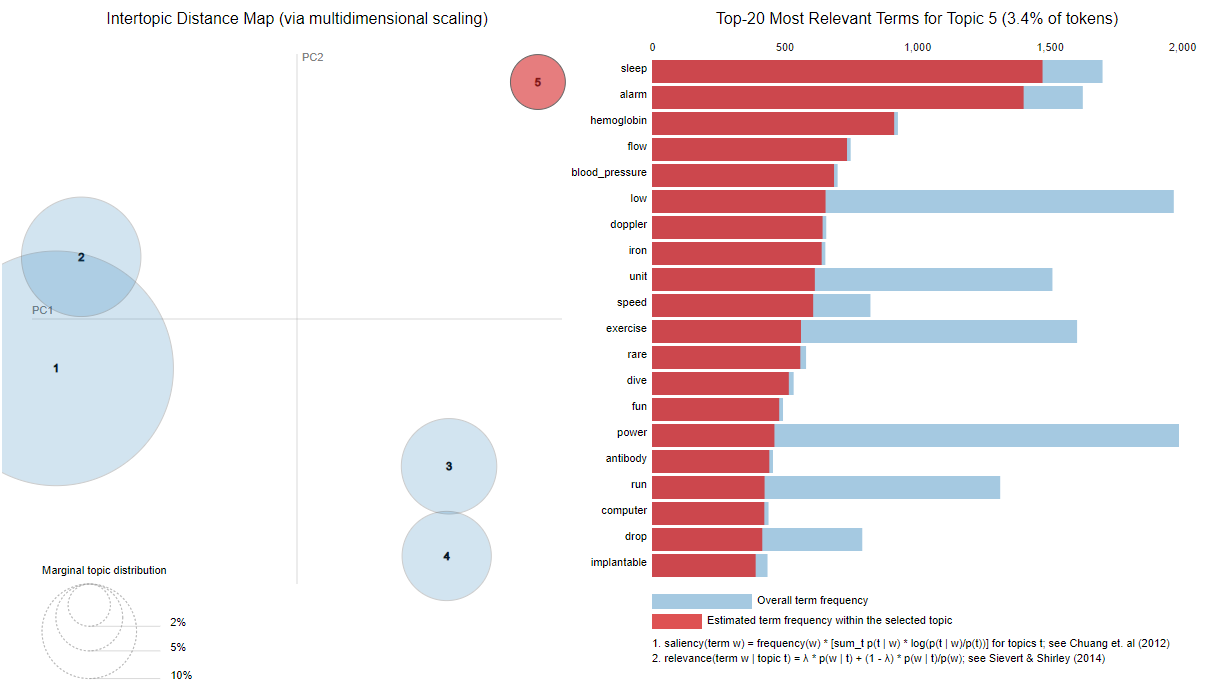


Stopwords

'i',

'me',

'my',

'myself',

'we',

'our',

'ours',

'ourselves',

'you',

"you're",

"you've",

"you'll",

"you'd",

'your',

'yours',

'yourself',

'yourselves',

'he',

'him',

'his',

'himself',

'she',

"she's",

'her',

'hers',

'herself',

'it',

"it's",

'its',

'itself',

'they',

'them',

'their',

'theirs',

'themselves',

'what',

'which',

'who',

'whom',

'this',

'that',

"that'll",

'these',

'those',

'am',

'is',

'are',

'was',

'were',

'be',

'been',

'being',

'have',

'has',

'had',

'having',

'do',

'does',

'did',

'doing',

'a',

'an',

'the',

'and',

'but',

'if',

'or',

'because',

'as',

'until',

'while',

'of',

'at',

'by',

'for',

'with',

'about',

'against',

'between',

'into',

'through',

'during',

'before',

'after',

'above',

'below',

'to',

'from',

'up',

'down',

'in',

'out',

'on',

'off',

'over',

'under',

'again',

'further',

'then',

'once',

'here',

'there',

'when',

'where',

'why',

'how',

'all',

'any',

'both',

'each',

'few',

'more',

'most',

'other',

'some',

'such',

'no',

'nor',

'not',

'only',

'own',

'same',

'so',

'than',

'too',

'very',

's',

't',

'can',

'will',

'just',

'don',

"don't",

'should',

"should've",

'now',

'd',

'll',

'm',

'o',

're',

've',

'y',

'ain',

'aren',

"aren't",

'couldn',

"couldn't",

'didn',

"didn't",

'doesn',

"doesn't",

'hadn',

"hadn't",

'hasn',

"hasn't",

'haven',

"haven't",

'isn',

"isn't",

'ma',

'mightn',

"mightn't",

'mustn',

"mustn't",

'needn',

"needn't",

'shan',

"shan't",

'shouldn',

"shouldn't",

'wasn',

"wasn't",

'weren',

"weren't",

'won',

"won't",

'wouldn',

"wouldn't"]
