# Supplementary material for: Using Topic Modeling to Understand Patients’ and Caregivers’ Perspectives About Left Ventricular Assist Device: Thematic Analysis
Source: J Med Internet Res. 2024 Aug 13;26:e50009. doi: 10.2196/50009 (PMC11350299; doi:10.2196/50009)
Supplement: Multimedia Appendix 2 [file jmir_v26i1e50009_app2.docx]

LDA Configuration:

gensim.models.LdaMulticore     (num_topics=5,

                                           passes = 10,

                                          iterations = 400,

                             workers=None,

                               chunksize=2000,

                               batch=False,

                               alpha='symmetric',

                               eta=None,

                               decay=0.5,

                               offset=1.0,

                               eval_every=10,

                               gamma_threshold=0.001,

                               random_state=None)
